# Long-term trajectories of BMI and cumulative incident metabolic syndrome: A cohort study

**DOI:** 10.3389/fendo.2022.915394

**Published:** 2022-12-08

**Authors:** Ming Ying, Xiangming Hu, Qiang Li, Haojian Dong, Yingling Zhou, Zhujun Chen

**Affiliations:** ^1^ Department of Cardiology, Fuwai Hospital Chinese Academy of Medical Sciences, Shenzhen, China; ^2^ Department of Cardiology, Guangdong Cardiovascular Institute, Guangdong Provincial People’s Hospital, Guangdong Academy of Medical Sciences, Guangzhou, China

**Keywords:** cohort study, metabolic syndrome, trajectory, obesity, body mass index

## Abstract

**Background:**

Body mass index (BMI) has been widely recognized as a risk factor for metabolic syndrome (MetS). However, the relationship between the trajectory of BMI and cumulative incident MetS is still unclear. We investigate the associations of long-term measurements of BMI with MetS among young adults in the China Health and Nutrition Survey.

**Methods:**

We enrolled individuals aged 10 to 20 at baseline with recorded BMI at each follow-up interview, and 554 participants were finally included in our study. The assessment and incidence of MetS were evaluated by blood tests and physical examinations in their adulthood. A latent class growth mixed model was used to identify three BMI trajectory patterns: a low baseline BMI with slow development (low-slow, n=438), a low baseline BMI with fast development (low-fast, n=66), and a high baseline BMI with fast development (high-fast, n=50). Logistic regression was used to explore the relationship between different BMI trajectories and the incidence of MetS.

**Result:**

During a follow-up of 16 years, 61 (11.01%) participants developed MetS. The combination of elevated triglycerides and reduced high-density lipoprotein cholesterol was most frequent in diagnosed MetS. In multivariate adjusted models, the low-fast and high-fast BMI trajectories showed a significantly higher risk of MetS than those with the low-slow BMI trajectory (low-high: OR = 3.40, 95% CI: 1.14-10.13, P < 0.05; high-fast: OR = 5.81, 95% CI: 1.63-20.69, P < 0.05).

**Conclusion:**

Our study identified three BMI trajectories in young adults and found that long-term measurements of BMI were also associated with cumulative incident MetS.

## Introduction

Metabolic syndrome (MetS), a common metabolic disorder syndrome, occurs in approximately 23% US population aged over 20 years old to 60% US population aged over 40 years old and its incidence has increased in developed countries (1–3). MetS is associated with an increased risk of coronary artery disease, cerebrovascular disease, and all-cause mortality (4). In fact, these diseases occur in adulthood and have certain correlations with metabolic disorders present in early adulthood that are overlooked (5, 6).

Obesity is a widely used indicator that represents nutrition excess, which contributes directly to metabolic disorders and thus leads to MetS, especially in the growth development period (7, 8). The relationship between abdominal obesity, defined based on waist circumference, and MetS was widely reported. Body mass index (BMI), used to measure general obesity, was found to have a relationship with MetS suggesting that exposure to adiposity may affect metabolism throughout the life course (6, 9). BMI baseline and patterns during early childhood all contribute to high risk of being overweight or obese in adulthood (10, 11). Previous research focused on the trajectory of BMI and found that change of BMI from 5 to 20 years old also has an important facilitation effect on the development of diseases, such as high carotid intima-media thickness, type 2 diabetes and cardiovascular disease (12–16). Regarding MetS, notably, previous studies frequently adopted single baseline measures of BMI while ignoring variations in BMI over time.

Although obesity plays a key role in the development of MetS, evidence of long-term obesity measurements and cumulative incident MetS is scarce. In this study, we used data from the China Health and Nutrition Survey (CHNS) to identify BMI trajectories derived from records of growth period and investigate the associations between BMI trajectories and cumulative incident MetS. As a simple and accessible index, BMI is gradually understood by Chinese parents. BMI trajectory, a trend formed by multiple measurements of BMI during adolescence, can be used as an efficient mean to monitor risk of MetS in further.

## Methods

### Study population

This study used data from CHNS, an ongoing population-based cohort study carried out by the government of China. We didn’t adopt the data collected in more recent than 2009, because these data don’t include blood sample, which is necessary to evaluate the MetS. From 1993 to 2009, six waves of surveys were completed in nine provinces (Liaoning, Jiangsu, Henan, Hunan, Guizhou, Heilongjiang, Shandong, Hubei and Guangxi) using a multistage, random-clustering process, with more than 12,000 participants enrolled. The study takes into account Chinese geographic distribution, economic development level and public health resources, and the sample can be considered to provide a representative data set reflecting ordinary people. The survey collected comprehensive demographic data, including sex, age, education level, dietary and nutritional status, health behavior and clinical data. Each participant signed an informed consent form. The relevant information related to this study has been published elsewhere (17).

**Figure 1:**
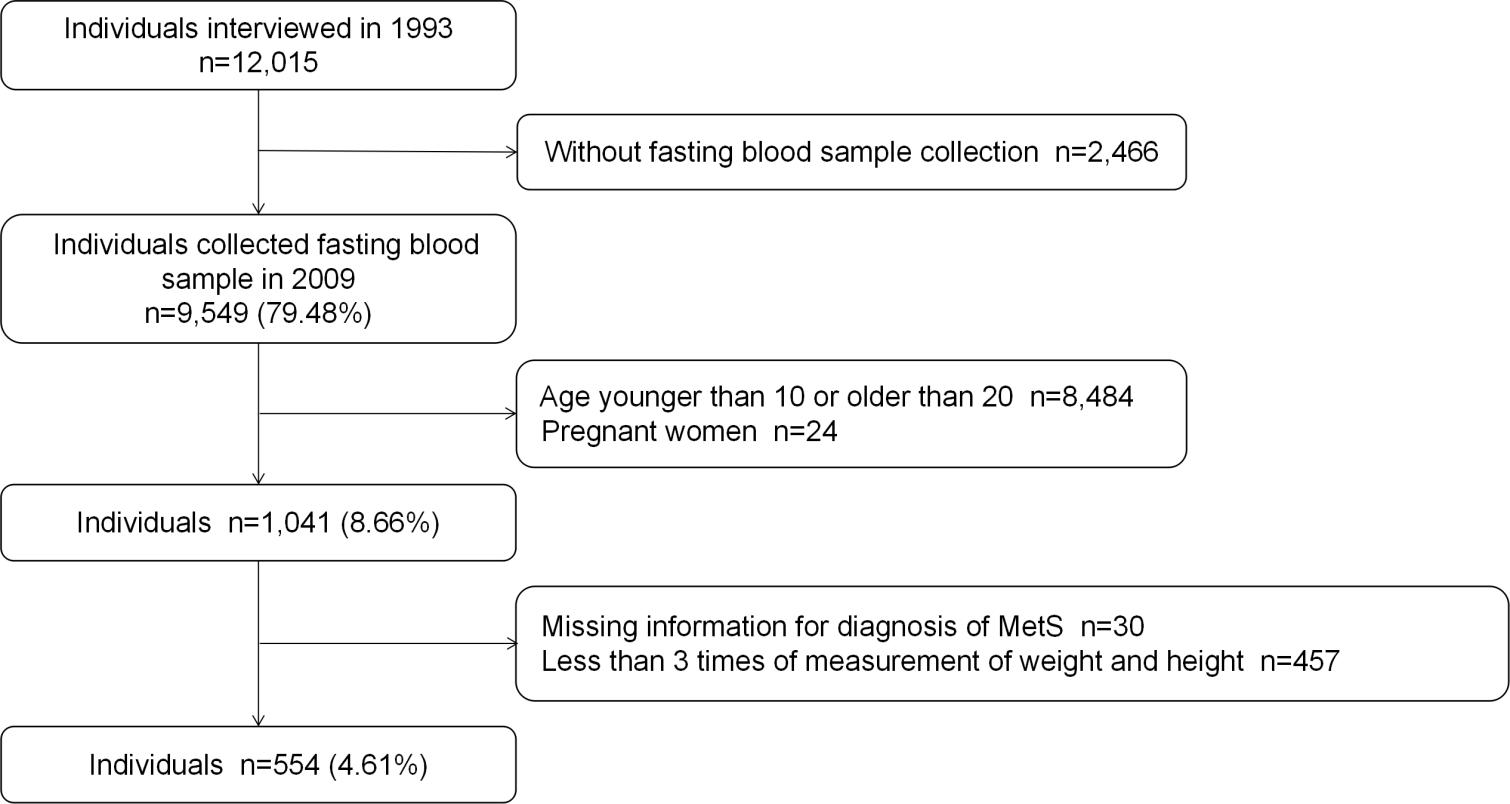
Study flow chart.

There were 12,015 participants enrolled in the CHNS study in 1993. The study staff conducted face-to-face interviews with participants in 1997, 2000, 2004, 2006 and 2009 to measure height and weight to calculate BMI. In 2009, 2,466 participants were excluded for missing fast blood example. We also excluded individuals who were aged < 10 years or > 20 years (n = 8,484) in 1993 at baseline, pregnant women (n = 24), people missing data to evaluate MetS according to the International Diabetes Federation criteria (n = 30) and people whose weight and height measurements were conducted less than 3 follow-up visits (n = 457) (**Figure 1**).

### Outcome

In 2009, all participants were adults aged 26-36, and MetS was evaluated in 2009 according to the International Diabetes Federation criteria (18), which was defined as central obesity (waist ≥ 90 cm in men or ≥ 80 cm in women) plus any two of the following: 1) elevated triglyceride (TG) (> 1.7 mmol/L) or specific treatment for TG abnormality; 2) reduced high-density lipoprotein cholesterol (HDL-C) (in men < 1.0 mmol/L or in women < 1.3 mmol/L) or specific treatment for HDL-C abnormality; 3) elevated blood pressure (SBP ≥ 130 mmHg or DBP ≥ 85 mmHg) or diagnosed hypertension; and 4) elevated fasting blood glucose (FBG) (≥ 5.6 mmol/L) or diagnosed type 2 diabetes mellitus (DM).

### Measurement and definition

Height and weight were measured while the subjects were wearing light clothing and no shoes. Blood collection and examination were performed by professional study staff. All subjects provided 12 mL of blood (in three 4-mL test tubes) after an overnight fast. The levels of HDL-C, TG, and FBG were assessed using a Hitachi 7,600 machine (Randox, Crumlin, UK; Kyowa, Tokyo, Japan). HbA1c was measured using an HLC-723 G7/D10/PDQ A1c Automated Glycohemoglobin Analyzer (Tosoh Bioscience LLC, Osaka, Japan; Bio-Rad Laboratories, Hercules, CA, USA; Primus Electronics, Morris, IL, USA). Intakes of total energy, carbohydrates, fat and protein were all calculated from participants’ average 3-day dietary intake data obtained by questionnaire (19). According to the American Diabetes Association criteria, DM was defined as previous DM history or FBG ≥ 7.0 mmol/L and/or HbA1c>= 6.5% (20). Waist circumference was measured with tape located at the level of the umbilicus when the participants stood normally with their feet 25-30 cm apart and at the end of the expiratory phase. Educational level and history of hypertension were obtained through self-report. Smoking was defined as current smoking (yes or no). Alcohol consumption was defined as drinking alcohol > 3 times/week (yes or no).

### Statistical analysis

For continuous variables, Student’s *t* test (for normally distributed variables)/Mann-Whitney U test (for variables with a skewed distribution) and analysis of variance (for normally distributed variables)/Kruskal-Wallis H (for variables with a skewed distribution) were used to detect differences between groups. For categorical variables, the chi-square test/Fisher’s exact test was used to detect differences between groups.

A latent class growth mixed model (LCGMM) was used to identify different trajectory patterns of BMI since it can take into account random individual variation and within-group variance. Based on the BMI measured three times or more by the participants, a growth model was established according to the measurements over time, while sex was taken as a covariate. Multiple LCGMMs were analyzed with different trajectories to obtain linear and nonlinear model parameters. The model selection criterion was based on the Bayesian information criterion, Vuong-Lo-Mendell-Rubin likelihood ratio test, efficiency of classification and posterior probabilities. After that, the estimated slope and variance in different trajectory classifications were obtained. P < 0.05 (two-sided) indicated statistical significance.

We explored the relationship between different BMI development trajectories and cumulative incident MetS through logistic regression to calculate the odds ratios (ORs) with 95% confidence intervals (CIs), and the effects of age, sex, BMI, waist circumference, residence, education background, smoking status, alcohol consumption, and nutritional intake were adjusted according to different models. All of the analyses were performed with Mplus 8, Stata 15.0 and R (version 3.6.1).

## Results

### Participant characteristics

**Figure 2:**
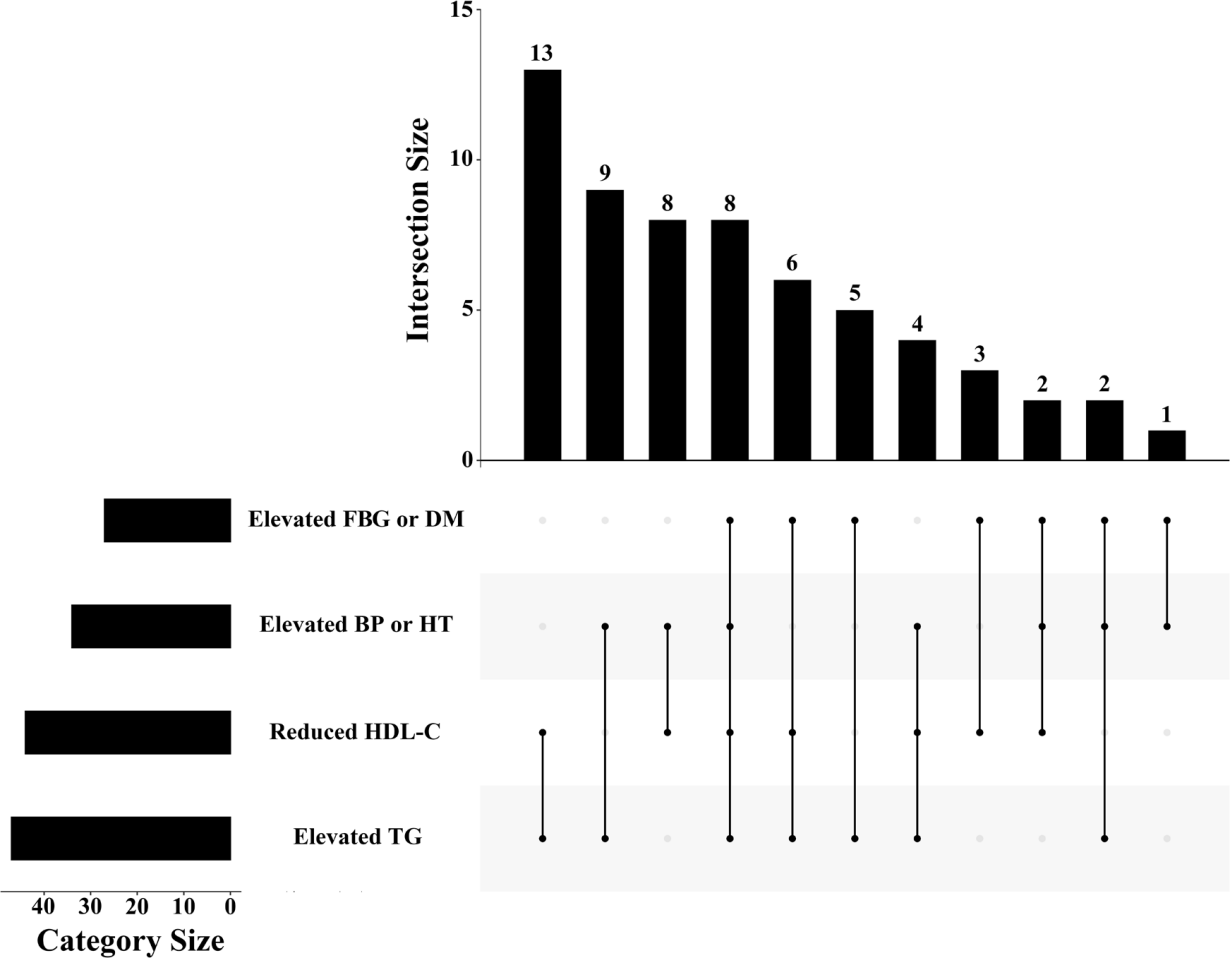
The distribution of diagnostic criteria among MetS patients in the study. FBG: fasting blood glucose; DM: diabetes mellitus; BP: blood pressure; HT: hypertension; HDL-C: high-density lipoprotein cholesterol; TG: triglyceride.

Of the 554 people without diabetes at baseline, 61 (11.0%) developed MetS during the 16-year follow-up. The details of meeting the diagnostic criteria of every MetS patient are shown in **Figure 2**. The combination of elevated TGs and reduced HDL-C was most frequent in diagnosed MetS. The baseline characteristics of the individuals and their follow-up characteristics as adults are shown in **Table 1**. People with MetS were older and had higher BMI and waist circumference. Concerning blood tests, patients with MetS were more likely to have abnormalities in blood lipids and blood sugar. Sex, residence, smoking and alcohol consumption were not significantly different between the two groups.

**Table 1:** Baseline characteristics by cumulative incident MetS at follow-up.

	Non-MetS(n=493)	MetS(n=61)	P-value
Age, year	31.9 ± 3.2	33.4 ± 2.7	<0.001
Female, %	227 (46.0%)	26 (42.6%)	0.613
Urban resident, %	162 (32.9%)	21 (34.4%)	0.806
Waist, cm	77.2 ± 8.6	94.8 ± 6.5	<0.001
BMI, kg/m^2^	22.0 ± 2.9	28.0 ± 2.7	<0.001
TG, mmol/L*	1.26 ± 0.88	3.43 ± 3.30	<0.001
HDL-C, mmol/L*	1.4 ± 0.4	1.0 ± 0.2	<0.001
FBG, mmol/L*	4.9 ± 0.6	5.8 ± 1.9	<0.001
Smoking, %	164 (33.27%)	23 (37.70%)	0.489
Alcohol consumption, %	235 (47.67%)	37 (60.66%)	0.056

Data are expressed as mean ± SD or N (%). Smoking was defined as previous smoking. Alcohol consumption was defined as drinking alcohol > 3 times/week

*Data was shown after interpolation by mean.

BMI: body mass index; TG: triglyceride; HDL-C: high-density lipoprotein cholesterol; FBG: fasting blood glucose; MetS: metabolic syndrome

### Trajectory analysis

According to the model selection criteria mentioned above, we modeled the BMI change tendency of each individual, compared the parameters of different class models **(** **Supplementary Table 1** **)** and finally chose a three-class model as the best fit. We numbered them Class 1 (low—slow), Class 2 (low—fast), and Class 3 (high—fast). Estimated and observed mean values of BMI are presented in **Supplementary Figure 1**. The estimated parameters of each model, including slope, intercept, quadratic parameters, and the incidence of MetS, are listed in **Table 2**. Except for the quadratic parameters in Class 2, the other three latent-variable parameters of the model were all positively correlated with MetS risk.

**Table 2:** Estimated mean of slope, intercept, and quadratic parameters for each class, and the incidence of MetS.

Classes	Slope, *P*	Intercept, *P*	Quadratic, *P*	Incidence of MetS
1	1.433, < 0.001	18.477, < 0.001	-0.176, < 0.001	10/438
2	1.447, 0.001	18.959, < 0.001	0.021, 0.791	22/66
3	2.454, < 0.001	23.314, < 0.001	-0.299, < 0.001	29/50

### Effects of BMI trajectory on the risk of MetS

**Figure 3:**
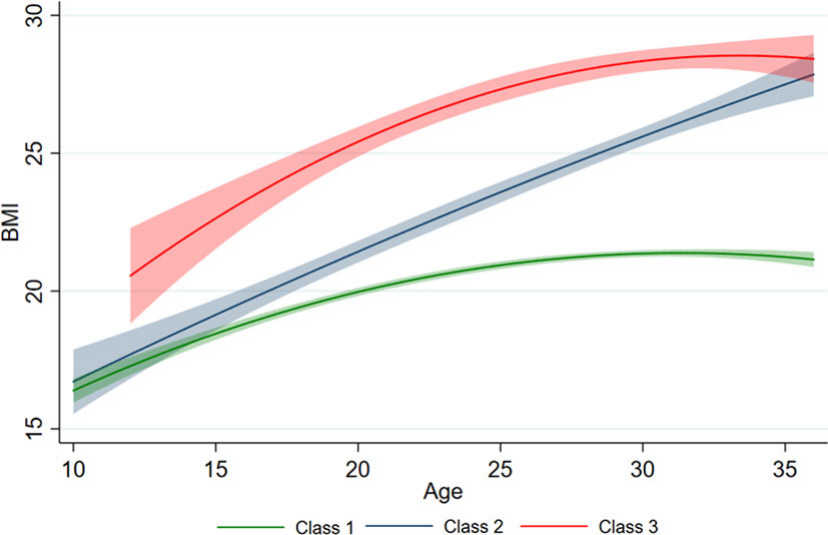
Different BMI trajectories developed by measurements of BMI. The predicted trajectory of BMI with age is presented by the solid line, and 95% of the CI is represented by shading.

**Figure 3** illustrates the longitudinal trajectory of the BMIs of adolescents aged 10–20 years with a low—slow trajectory (Class 1, 79.1%, n = 438), a low—fast trajectory (Class 2, 11.9%, n = 66), and a high—fast trajectory (Class 3, 9.0%, n = 50). Class 1 shows a steady growth trend, while Class 2 shows a rapid growth pattern with a low baseline value. Class 3 presented a steady growth pattern with a high baseline value. By 2009, the BMI values of Class 2 and Class 3 were close. At an early age, BMI increased faster (positive slope) in all three classes. In early adolescence, classes 1 and 3 tended to be stable, while class 2 continued to increase faster.

Participants were also grouped according to changes in BMI (**Table 3**). We discovered that participants in Class 3 were inclined to have higher BMI, waist circumference, blood lipids and blood sugar at follow-up. Moreover, the change in BMI showed a significant difference between the sexes. The BMI of Class 2 increased faster in both men and women. However, in Class 3, it seems that the rapid increase in early youth was attributed to males (**Supplementary Figure 2**).

**Table 3:** Baseline characteristics by different classes at follow-up.

BMI trajectory	1(n = 438)	2(n = 66)	3(n = 50)	P-value
Age, year	32.0 ± 3.2	31.7 ± 3.2	33.1 ± 2.4	0.051
Female, %	214 (48.9%)	14 (21.2%)	25 (50.0%)	<0.001
Waist, cm	75.8 ± 7.7	75.8 ± 7.7	93.4 ± 6.8	<0.001
BMI, kg/m^2^	21.3 ± 2.1	27.4 ± 1.9	28.4 ± 2.5	<0.001
Urban resident, %	141 (32.2%)	25 (37.9%)	17 (34.0%)	0.650
Average energy intake, kcal/day†	2090.4 (1752.0-2574.9)	2453.6 (1954.3-2836.0)	2226.1 (1851.6-2800.7)	0.007
Average carbohydrate intake, g/day†	295.1 (235.8-375.8)	335.4 (310.7-397.0)	291.1 (241.5-342.2)	0.015
Average fat intake, g/day†	68.9 (50.1-92.9)	80.0 (53.6-105.5)	79.57 (65.2-103.6)	0.006
Average protein intake, g/day†	65.2 (53.0-80.6)	72.1 (62.0-92.3)	69.1 (52.6-97.3)	0.020
TG, mmol/L*	1.25 ± 0.87	2.46 ± 3.03	2.46 ± 2.14	<0.001
HDL-C, mmol/L*	1.4 ± 0.4	1.1 ± 0.2	1.2 ± 0.3	<0.001
FBG, mmol/L*	4.9 ± 0.6	5.3 ± 0.8	5.8 ± 2.1	<0.001
Level of education, %				0.908
Primary school or below	19 (4.3%)	2 (3.0%)	2 (4.0%)	
Graduated from primary school	61 (13.9%)	9 (13.6%)	8 (16.0%)	
Lower middle school degree	218 (49.8%)	33 (50.0%)	23 (46.0%)	
Upper middle school degree	45 (10.3%)	9 (13.6%)	9 (18.0%)	
Technical or vocational degree	58 (13.2%)	7 (10.6%)	6 (12.0%)	
University or college degree	37 (8.4%)	6 (9.1%)	2 (4.0%)	
Smoking, %	147 (33.6%)	25 (37.9%)	15 (30.0%)	0.662
Alcohol consumption, %	202 (46.1%)	43 (65.1%)	27 (54.0%)	0.012
MetS, %	10 (2.3%)	22 (33.3%)	29 (58.0%)	<0.001
Central obesity	54 (12.33%)	45 (68.18%)	48 (96.00%)	<0.001
Elevated TG	90 (20.55%)	32 (48.48%)	26 (52.00%)	<0.001
Reduced HDL-C	78 (17.81%)	30 (45.45%)	24 (48.00%)	<0.001
Elevated BP or HT	57 (13.01%)	21 (31.82%)	21 (42.00%)	<0.001
Elevated fasting blood glucose or DM	37 (8.45%)	17 (25.76%)	14 (28.00%)	<0.001

Data are expressed as mean ± SD or median (Q1–Q3) or N (%). Smoking was defined as previous smoking. Alcohol consumption was defined as drinking alcohol > 3 times/week

†Data was shown after interpolation by median.

*Data was shown after interpolation by mean.

BMI: body mass index; TG: triglyceride; HDL-C: high-density lipoprotein cholesterol; FBG: fasting blood glucose; BP: blood pressure; HT: Hypertension; DM: diabetes mellitus; MetS: metabolic syndrome.

**Figure 4:**
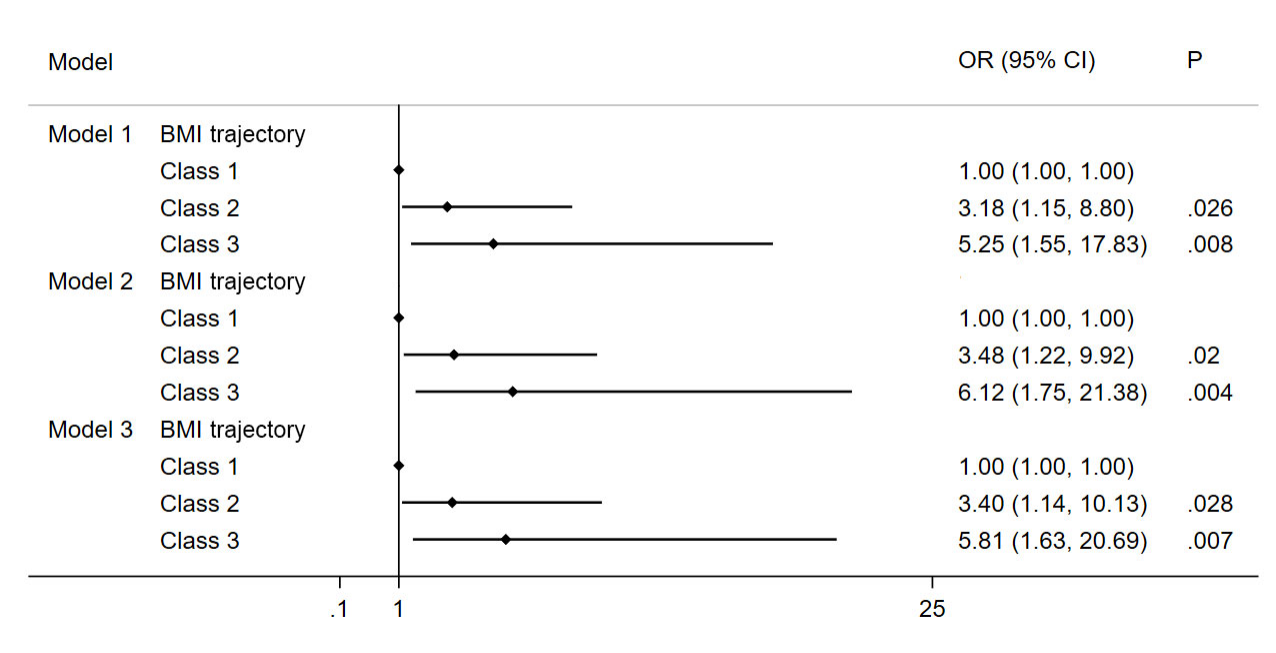
Association of MetS and BMI trajectory in the study subjects. Note: Model 1 Adjusted for age, gender, BMI and waist circumference; Model 2 Adjusted for Model 1 and education background, residence, smoker, alcohol consumption; Model 3 Adjusted for Model 2 and energy intake.

The results of the multivariate logistic regression model showed that different trajectory patterns were associated with the occurrence of MetS in adolescents after adjustment for variables (**Figure 4**). In the fully adjusted models, participants in Class 2 and Class 3 had a significantly higher risk of MetS than Class 1 participants (Class 2: OR = 3.40, 95% CI: 1.14—10.13, P < 0.05; Class 3: OR = 5.81, 95% CI: 1.63—20.69, P < 0.05).

## Discussion

Our study identified three types of BMI trajectories in early adults, and the risks of cumulative incident MetS were different according to these trajectories. Individuals with a low—fast or a high—fast BMI trajectory had a higher risk of MetS in early adults than those with a low—slow trajectory, regardless of the initial BMI level. Adjustment of the models for several relevant demographic and cardiometabolic variables did not dampen their associations.

This study provided new evidence for the relationship between BMI and metabolic disorders in several aspects. The study focuses on teenagers who were in the growth period, and the changes in BMI can be sensitively captured to reflect machine metabolism. Then, the participants with MetS in the study commonly had increased TGs and decreased HDL-C, which suggested that lipid disorder was the culprit of MetS development in those patients. In addition, we also found that the rapid increase in BMI in teenagers was attributed more to males than females in China. Young men were more likely to be exposed to rapidly increasing BMIs. Finally, long-term measurements of BMI that were prospectively recorded for 16 years were used to validate the relationship between the BMI trajectory and the incidence of MetS.

The relationship between obesity and MetS has been widely explored. Ofer et al. performed a study enrolling 23,993 individuals and found that normal BMI had a high negative predictive value for MetS and pointed out that BMI equal to 27 was the ideal value for the identification of MetS in the entire cohort (21). Stolzman et al. discovered that adolescents with higher BMI demonstrated a greater incidence of MetS than those with normal BMI (22). Teenage adiposity is a good predictor of MetS in adults, especially in those with rapid growth of adipose tissue (23, 24). Although BMI as an indicator of adiposity is closely related to the occurrence and development of MetS, a one-time measurement does not represent changes in BMI over time. Another study including 5,317 university graduates with a median follow-up time of 6.1 years concluded that weight gain from childhood to adolescence/young adulthood increased the chance of cumulative incident MetS in adulthood, which was also consistent with our findings. However, there were some limitations that may cause controversy. First, the research used participant body images at aged 5 and aged 20 to estimate the change in BMI, which may have led to inaccurate information on the change in BMI. Second, the study was conducted using a questionnaire, and the conclusion of the study was greatly affected by selection bias. Recently, Liu et al. conducted a similar study among 93 students aged between 18 and 22 years in Liaoning Province in China (25). The participants were divided into four study groups according to the baseline level of BMI and change in BMI values. They found that both baseline BMI and BMI changes were predictive of MetS in early adulthood and that adolescents with a baseline BMI > 23.47 kg/m^2^ or a change in BMI > 1.95 kg/m^2^ were at increased risk of MetS in adulthood. Our study fitted the BMI trajectory of teenagers based on a longitudinal study in nine provinces for 16 years, which was representative and better classified different classes for screening those with a high risk of MetS. The association between BMI trajectory and cumulative incident MetS was independent of baseline BMI and demographic characteristics such as age, sex, and education level. The change in BMI represents the accumulation of adiposity, which can better reflect the degree of individual exposure to obesity over time.

Currently, although it is generally recognized that obesity and insulin resistance are the central link of MetS, the mechanism of the association between changes in BMI and MetS is not entirely clear. Body fat content and fat distribution are considered important indicators of health status (26). Central accumulation of body fat accompanied by an increase in BMI is associated with insulin resistance (27). Insulin resistance occurs when glucose uptake and utilization are impaired and is characterized by metabolic disorders such as decreased glucose tolerance, hyperinsulinemia, increased TG and decreased HDL-C (28). After knocking out insulin receptors in mice, adipose tissue is rapidly consumed due to increased lipolysis and adipocyte apoptosis, which leads to insulin resistance and glucose intolerance (29). The discovery of endocrine and immune properties of adipocytes has provided further mechanistic insights into the development of MetS. Adiponectin was found to protect against the development of hypertension, diabetes, and acute coronary syndrome (30, 31). Moreover, activation of the renin-angiotensin system serves as an important neurohumoral pathway of obesity contributing to the development of MetS. Obesity and insulin resistance are associated with increased production of angiotensin II, which can activate nicotinamide adenine dinucleotide phosphate oxidase, leading to the generation of reactive oxygen species (32). These studies focus on the etiology of MetS, supporting that obesity is a dynamic evolution process of continuous adipose accumulation at the molecular level. The BMI trajectory not only represents changes in body shape but, more importantly, changes in various nutrient metabolism pathways in the organism, which are closely related to the pathogenesis of MetS. We also found that lipid disorders contributed mostly to MetS diagnosis in early adulthood.

The results from this study may have an impact on guidelines for MetS prevention and management. Previous study reported that obesity in adulthood significantly further aggravated the risk of MetS in individuals who experienced early life undernutrition (33). While, children face the serious problem of overnutrition and overweight in future. It’s meaningful to clear the model of children obesity. Public health emphasis on the control of long-term BMI and dietary management in teenagers, especially in men, may effectively reduce the incidence of MetS in the population.

Our study also has limitations. First, as the CHNS encompassed children to older adults, we extracted adolescents from CHNS to establish the cohort. Although this approach is not as good as direct recruitment of adolescents, it can still represent the general individuals to some extent in the context of the rigorous study design and random cluster sampling process of CHNS. Second, the sample size of this study was relatively small, but our pilot study provides a new insight into the effect dynamic change of BMI on MetS, which could provide the perspective for future large sample studies. Third, because CHNS did not collect blood samples when participants were enrolled, we could not identify those with diagnosed MetS at baseline and exclude them. Nevertheless, based on the study results, the incidence of MetS was low, and the initial MetS would unlikely be high, so its impact could be relatively small. Finally, the information of lifestyle of the subjects was unavailable. It should be noted that to be sedentary or active with moderate or vigorous intensity affect the metabolism and the development of MetS.

## Conclusion

Our results identify three BMI trajectories in Chinese teenagers based on a 16-year longitudinal study. BMI trajectories of low—fast and high—fast are associated with an increased incidence of MetS in early adulthood, and young men were more likely to be exposed to rapidly increasing BMIs. We also found that lipid disorders contributed mostly to MetS diagnosis in early adulthood.

## Data availability statement

Publicly available datasets were analyzed in this study. This data can be obtained from https://www.cpc.unc.edu/projects/china.

## Ethics statement

Study protocols were approved by the Institutional Review Committees of the University of North Carolina at Chapel Hill, USA, and the National Institute for Nutrition and Health (NINH, former National Institute of Nutrition and Food Safety) at the Chinese Center for Disease Control and Prevention (CCDC) at Beijing, China.

## Author contributions

MY: Conceptualization, data curation, formal analysis, writing-original draft, writing-review and editing. XH: Data curation, writing-review and editing. QL and HD: Writing-review and editing. YZ and ZC: Conceptualization, writing - review and editing and funding acquisition. All authors contributed to the article and approved the submitted version.

## Supplementary material

Supplementary Figure 1 Estimated and observed mean AVI values

Supplementary Figure 2 The predicted trajectory of AVI with age in different genders is presented by the solid line, and 95% of the CI is represented by shading
