# Supplementary figures and images for: Long-term trajectories of BMI and cumulative incident metabolic syndrome: A cohort study
Source: Front Endocrinol (Lausanne). 2022 Dec 8;13:915394. doi: 10.3389/fendo.2022.915394 (PMC9773063; doi:10.3389/fendo.2022.915394)

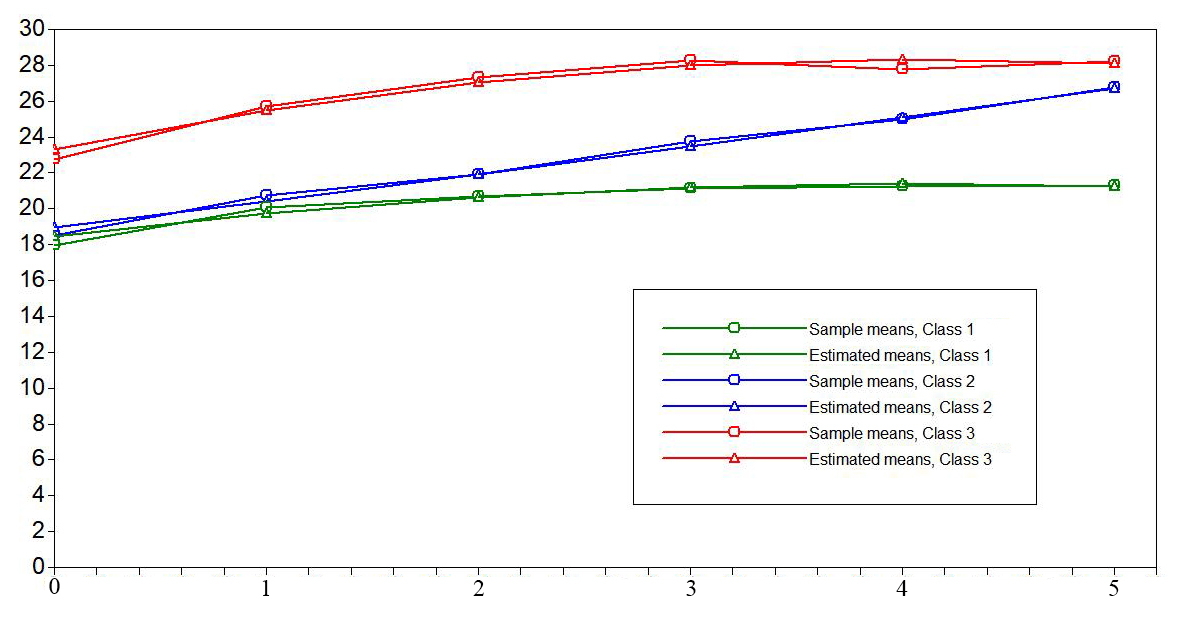

Supplement: Supplementary Figure 1 — Estimated and observed mean AVI values [file Image_1.png]

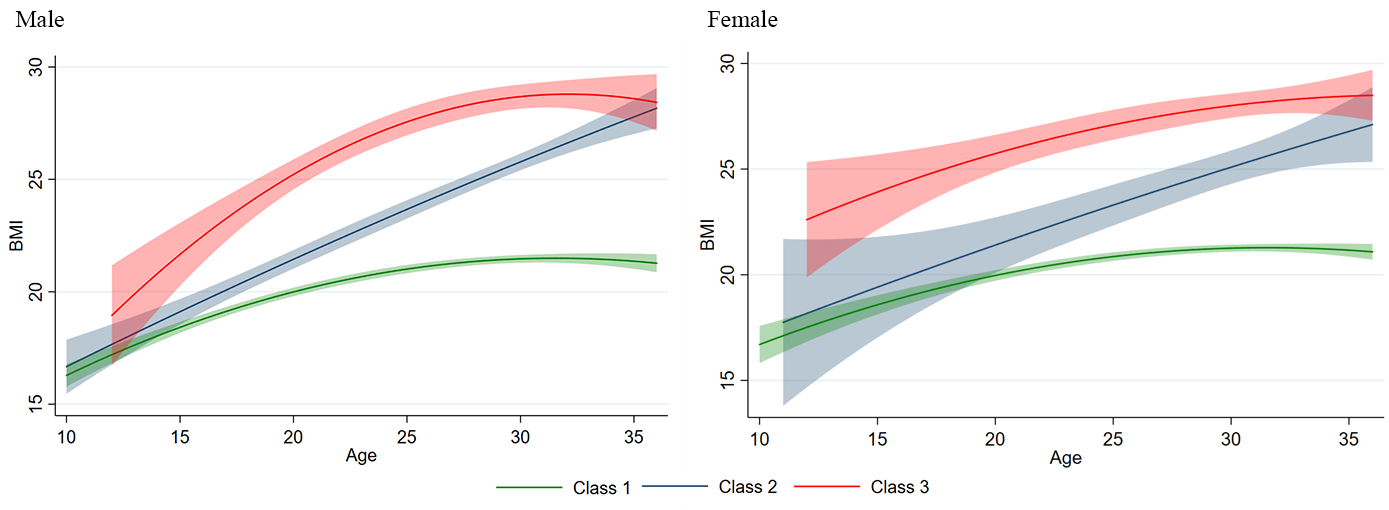

Supplement: Supplementary Figure 2 — The predicted trajectory of AVI with age in different genders is presented by the solid line, and 95% of the CI is represented by shading [file Image_2.png]
