# Supplementary material for: Long-term trajectories of BMI and cumulative incident metabolic syndrome: A cohort study
Source: Front Endocrinol (Lausanne). 2022 Dec 8;13:915394. doi: 10.3389/fendo.2022.915394 (PMC9773063; doi:10.3389/fendo.2022.915394)
Supplement: Supplementary file 3 [file Table_1.docx]

**Supplementary Table S1.** Fitting parameters and results of different classifications in the latent class growth mixed model

| Classes | Polynomial formula | Entropy* | *P*-value† | BIC | Class proportion, % | Average latent class probabilities, % |
| --- | --- | --- | --- | --- | --- | --- |
| 2 | Linear | 0.853 | 0.0098 | 9778.684 | 90.6/9.4 | 97.2/83.5 |
|  | Quadratic | 0.868 | 0.0012 | 9678.031 | 7.8/97.2 | 85.2/97.2 |
| 3 | Linear | 0.774 | 0.0489 | 9760.303 | 8.5/12.6/78.9 | 80.1/76.9/93.2 |
|  | Quadratic | 0.810 | 0.0219 | 9655.094 | 11.9/79.1/9.0 | 81.0/95.0/80.7 |
| 4 | Linear | 0.775 | 0.0668 | 9757.296 | 7.4/4.0/17.7/70.9 | 74.0/89.1/80.1/91.0 |
|  | Quadratic | 0.852 | 0.0768 | 9662.710 | 77.8/8.8/12.1/1.3 | 95.0/81.8/84.3/89.7 |

Note: BIC, Bayesian information criterion.

*Entropy is an index of categorical separation: it ranges from 0 to 1, and values of 0.8 or higher are considered a sign of a useful model.

†*P*-value obtained by Vuong–Lo–Mendell–Rubin, testing whether the number of classes provides an improved model fit compared to a model that uses fewer classes.
